# An Evaluation of the Efficacy of Selective Alpha-Blockers in the Treatment of Children with Neurogenic Bladder Dysfunction—Preliminary Findings

**DOI:** 10.3390/ijerph13030321

**Published:** 2016-03-15

**Authors:** Paweł Kroll, Ewa Gajewska, Jacek Zachwieja, Magdalena Sobieska, Przemysław Mańkowski

**Affiliations:** 1 Pediatric Surgery and Urology Department, Neurourology Unit, Poznań University of Medical Sciences, ul. Szpitalna 27/33, Poznań 60-572, Poland; mankowskip@wp.pl; 2 Chair for Rheumatology and Rehabilitation, Poznań University of Medical Sciences, ul. 28 Czerwca 135/147, Poznań 61-545, Poland; ewagajewska1011@gmail.com (E.G.); msobieska@ump.edu.pl (M.S.); 3 Pediatric Nephrology Department, Poznań University of Medical Sciences, ul. Szpitalna 27/33, Poznań 60-572, Poland; zachwiej@mp.pl

**Keywords:** neurogenic bladder dysfunction, alpha-blocker, doxazosin, sphincter, urodynamics

## Abstract

The aim of this study was to assess the usefulness of selective α1-blockers in children with neurogenic urinary tract dysfunctions and increased leak point pressure (LPP). 14 children from age 6 to 16 years with neurogenic urinary tract dysfunctions (neurogenic bladder) and LPP > 40 cm H_2_O were enrolled in the study. All patients received a selective α1-blocker (doxazosin) for 6–8 weeks with an initial dosage of 0.03 mg/kg. During the observation period the continuation of oral anticholinergics, Clean Intermittent Catheterization (CIC), observation of “urinary dryness” and urinary incontinence periods were recommended. Patients were scheduled for a follow-up visit and urodynamic investigation after 6–8 weeks after the doxazosin therapy was started. In 4 patients, urine leakage occurred at lower pressures; in 9 patients, no significant changes in urine leak point pressures were detected; in 3 patients, there was a significant increase in the bladder capacity; in one patient, deterioration in continence was noted. The differences both in LPP and LPV before and after the treatment were not statistically significant. Our observations are consistent with the conclusions from other studies and showed no evident efficacy of doxazosin in children with neurogenic bladder.

## 1. Introduction

Normal functioning of the bladder involves periodic urination planned by a patient in portions of the volume appropriate for the patient’s age. The term neurogenic bladder is used with reference to situations when abnormal functioning of the bladder is caused by known defects or nervous system diseases. A neurogenic bladder is found in children most frequently as a consequence of dysraphic defects and causes significant loss of the bladder fullness sensation and the sensation of the urethra. Consequently, a patient has no control over the function of the urinary tract, which manifests itself in the inability to urinate in one large portion on the one hand, and in incontinence on the other hand [1].

In patients with the aforementioned disorders, the treatment is based on periodic emptying of the bladder by Clean Intermittent Catheterization (CIC) and pharmacological reduction of the pressure in the bladder with subsequent enlargement of the bladder.

Oral anticholinergic drugs are administered in the pharmacotherapy of children with a high-pressure overactive neurogenic bladder. Anticholinergics decrease the pressure in the bladder in the storage phase. However, in the majority of children with a neurogenic bladder, increased sphincter tension is also observed in urodynamic investigations. In this group of patients, urine flows out of the bladder at high pressures if it is not removed from the bladder using CIC [1,2].

Detrusor leak point pressure (LPP) is the lowest value of detrusor pressure at which leakage from the bladder is observed during urodynamic evaluation. The term “hyperactive sphincter” is used with reference to urinary tract disorders manifested by a LPP greater than 40 cm H_2_O [3].

Patients with an elevated LPP and decreased bladder volume are more likely to develop serious complications such as recurrent urinary tract infections, or secondary anatomical changes in the urinary tract. Low bladder capacity with an increase of LPP to greater than 40 cm H_2_O reduces glomerular filtration rate, and leads to deterioration of ureteral drainage and hydronephrosios, which may result in renal insufficiency. Intervention to decrease LPP minimizes the risk of renal damage [1,3,4,5].

A decrease of LPP to lower than 40 cm H_2_O in association with an increase of bladder volume reduces the risk of complications development.

The use of α-blockers is aimed at decreasing tension of the urethral sphincter, reducing the functional subvesical obstruction and decresase of LPP.

There are some reports on the use of α-blockers both in patients with neurogenic and idiopathic bladder dysfunctions.

The authors of this study describe their own experiences in the application of α1-blockers in children with neurogenic bladder dysfunctions and hyperreactive sphincters in the aim to decrease LPP.

### Aim of the Study

The aim of this study is to assess the usefulness of selective α1-blockers to decrease LPP in children with neurogenic urinary tract dysfunctions accompanied by increased sphincter tension and elevated LPP.

## 2. Materials and Methods

14 children were enrolled in the prospective study. These were children with neurogenic urinary tract dysfunctions (neurogenic bladder). They showed elevated LPP in urodynamic examinations.

Due to bladder disorders all of the children had been treated conservatively with CIC. Additionally, all of these children were diagnosed with detrusor overactivity in urodynamics and were administered oral oxybutynin.

All patients underwent 3 consecutive cystometric examinations performed in a routine manner according to recommendations of International Children’s Continence Society (ICCS): double lumen catheter for measuring intravesical pressure and infusion, rectal tube for intraabdominal pressure, 0.9% NaCl saline in room temperature and infusion with a filling rate of 5%–10% of Expected Bladder Capacity (EBC). 

Infusion during cystometry was performed until urine leakage from the urethra (Leak Point) or complaints of urgency or bladder pain occurred. LPP and the volume at which the leakage occurred (leak point volume = LPV) were noted.

The inclusion criterion for the study was the diagnosis of an elevated LPP above 40 cm H_2_O in a child with neurogenic bladder dysfunction.

The patients were instructed to take a selective α1-blocker (doxazosin Table 1, 2 mg) in initial dosage 0.03 mg/kg. A precise dosage in mg/kg was not possible as tablets were used, so we proposed:

1. patients with body weight of up to 20 kg doxazosin 0.5 mg to 1 mg,
2. patients with body weight between 20 kg and 40 kg: doxazosin 1 mg to 2 mg, and
3. patients with body weight above 40 kg: 2 mg doxazosin in a single dose in the evening.

During the observation period, the continuation of oral anticholinergics, the continuation of CIC, checking the volume of the catheterized urine, observation of “urinary dryness” and urinary incontinence periods were recommended.

All patients were conducting “catheterization/voiding diary”.

Patients were scheduled for a follow-up visit and urodynamic investigation after 6–8 weeks after the α1-blocker therapy was started.

Statistical analysis was performed using the StatSoft, Inc. Polska Kraków, Poland (2011) software package STATISTICA (data analysis software system), version 10 (StatSoft Polska, Kraków, Poland).

Due to a small number of patients and the distribution of data being different from normal, the non-parametric statistical analysis was applied. As the group described was very heterogeneous, the results were expressed as median and quartiles, a sign test was used to compare the results before and after treatment and Spearman’s test was used to look for correlation between the variables.

### Ethical Statement 

All subjects gave their informed consent for inclusion before they participated in the study. The study was conducted in accordance with the Declaration of Helsinki, and the protocol was approved by the Local Ethics Committee for Poznań University of Medical Sciences (1221/06).

## 3. Results

The study included 14 children, 5 boys and 9 girls. The children were aged from 6 to 16 years.

Meningomyelocele was the cause of the neurogenic bladder dysfunction in all of them, but 1 had occult spinal dysraphisim. In 7 of them, hydronephrotic dilatation of the collecting system was estimated in ultrasonography. The anterio-posterior diameter of the renal pelvis ranged from 10 mm to 30 mm.

All children underwent urodynamic examinations. These examinations evaluated the sensation of the urethra and the bladder fullness sensation, leak point volume (LPV), and leak point pressure (LPP), and compliance and contractility of the bladder walls were determined.

In all children, increased LPP and decreased LPV were estimated in the cystometries.

In the study group, LPP amounted to 45–210 cm H_2_O (median: 98 Q25–Q75: 90–129 cm H_2_O), and LPV amounted to 30–290 mL (median: 135, Q25–Q75: 62–209 mL).

One boy who experienced side effects, drowsiness and a decrease in blood pressure failed to complete the study. None of the children developed symptomatic urinary tract infections during the study.

The follow-up urodynamic tests were performed in 13 patients after 6–8 weeks of treatment with the selective α1-blocker (doxazosin).

None of the patients manifested any changes in the sensation of the urethra or the bladder fullness.

In children treated with doxazosin, LPP amounted to 30–200 cm H_2_O (median: 90; Q25–Q75: 56–115 cm H_2_O), and LPV amounted to 30–280 mL (median: 145, Q25–Q75: 68–230).

In 4 patients, urine leakage occurred at marked lower pressures; in 9 patients, no significant changes in urine leak point pressures were detected.

In 3 patients, a significant increase in the bladder capacity, which was due to the decrease in detrusor overactivity, was noted. However, none of the patients reached expected bladder capacity.

**Figure 1:**
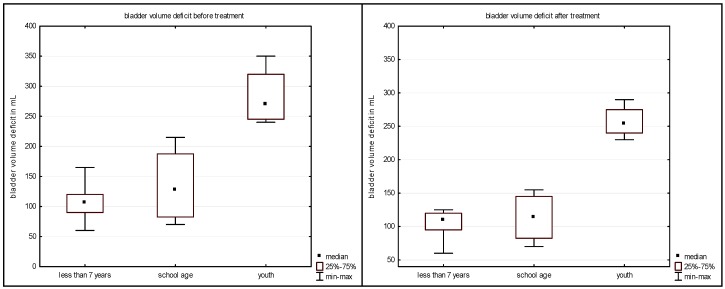
The difference between the expected and measured bladder volume in the investigated children.

Improvement by increase in bladder volume was seen mostly in older children, which is shown in Figure 1.

The differences both in LPP and in LPV before and after the treatment were not statistically significant (sign test, *p* = 0.113 and 0.505, respectively). Nevertheless, it could be seen that the deficit towards the due bladder volume was largest in the oldest patients’ group; additionally, in this group, the improvement was relatively largest.

One patient experienced deterioration of continence, and portions of the catheterized urine were also reduced. In 3 children, improvement of hydronephrosis was estimated in post-treatment ultrasonograpic evaluations. The data from urodynamic evaluations are shown in Table 1.

The statistically significant correlation was found between the patient’s age and LPP ((Spearman’ rho = 0.664, *p* < 0.05), as well as LPV (Spearman’ rho = 0.876).

There was no correlation between patient’s age and the change in LPP nor for LPV before and after the treatment. (Spearman’ rho = 0.066 for LPP (Spearman’ rho = −0.252 for LPV, *p* < 0.005).

## 4. Discussion

Conservative treatment, which consists of CIC and oral pharmacotherapy using anticholinergics, is a standard procedure in children with a neurogenic bladder dysfunction. Anticholinergic drugs cause both a reduction in the tonic tension of bladder muscles and its overactivity. This leads to reduced pressures inside the bladder during the storage phase and increase in the functional bladder capacity, which is expressed by the increase in the catheterized urine volume and prolonged urinary dryness periods between catheterization procedures. According to the data from many centers, in 70% of patients subjected to this type of treatment, no complications have been observed. Unsatisfactory results of conservative treatment are most frequently due to the persisting detrusor overactivity or sphincter hyperactivity, resulting in high pressures during urine leakage from the bladder [1,3,4,5,6,7].

In the past, patients with neurogenic bladder and sphincter hyperactivity underwent permanent bladder drainage using the Foley catheter (indwelling catheters), diversion procedures, vesicostomy and ureterocutaneostomy, and even sphincter incision [4,5].

Currently, the main treatment method in these patients is CIC [1].

In case of concomitant detrusor overactivity, anticholinergic pharmacotherapy is also recommended [1,6,7].

The sympathetic part of the autonomic nervous system is primarily responsible for urine continence in the bladder. In the sphincter and bladder neck muscles, prevalence of α1-andregenic receptors is found. Their activation increases the sphincter mechanism tension, and inactivation enables voiding [6,7].

The consequence of increased sphincter activity is the accumulation of urine in the bladder at high pressures and its leakage from the bladder only if the pressure exceeds values considered harmful to the kidneys [1,2,6,7].

Urodynamic examinations are essential for the assessment of the functioning of the lower urinary tract. What is of greatest significance in children with a neurogenic bladder is the determining the value of LPP. Following other authors, we assumed a LPP above 40 cm H_2_O to be considered increased [2].

The application of α-blockers is aimed at decreasing their excessive tension and reducing the functional subvesical obstruction. The application of α-blockers causes a reversible, periodic reduction of the activity of the α-adrenergic part of the autonomic nervous system.

Several preparations are available in the group of α-blockers: alfuzosin (Dalfaz), tamsulosin (Omnic) and doxazosin (Doxar).

The efficacy of selective α-blockers in patients with subvesical obstruction caused by benign prostatic hypertrophy is well documented [8,9].

Reports on successful α-blockers management in children with ureteral stones were also published [10,11].

There are few reports on the use of α-blockers in patients with neurogenic bladder.

In 2003 Abrams *et al.* showed efficacy and safety of tamsulosin treatment in patients with neurogenic lower urinary tract dysfunction due to suprasacral spinal cord injury [12].

Other studies have shown variable degrees of efficacy when using selective α-blockers in children with neurogenic and non-neurogenic bladder dysfunction [13,14,15,16,17,18,19,20].

Data from randomized trial on tamsulosin in 135 children with neurogenic bladder were very interesting: Although 51 (37.8%) patients were LPP “responders”, no statistically significant difference was found in LPP response rates between tamsulosin and placebo groups [19].

In an uncontrolled study on 17 children with neurogenic bladder from 2011 Schulte-Baukloh *et al.* showed that alfuzosin decreases the detrusor LPP significantly, and should be considered an alternative or addition to intermittent catheterization in selected patients [20].

Our data supports those findings: In some children with a neurogenic bladder, a marked decrease in LPP was observed, whereas no effect was observed in others.

In our opinion, their efficacy in patients with neurogenic bladders is hampered by the fact that the essence of the problem is the absence of central control of the function of urinary tract organs. This lack of control is responsible for both sphincter spasticity and detrusor contractile hyperreactivity.

Many irregularities related both to the quality, quantity and the function of receptors in the walls of the bladder and sphincters have been reported in patients with neurogenic urinary tract dysfunctions. The activity of other than α adrenergic receptors may also be explained by the inactivity of α-blockers in the majority of our patients [21,22,23,24].

Our observations relate to a relative small group of patients. This study was not randomized.

The drug was administrated “*off-label*,*”* according to data from other studies. The treatment was started with a dose of 0.03 mg/kg doxazosin [11,13,14,15].

An interesting observation seems to be the significant decrease in LPP corresponding to the sphincter function in four patients. In three patients, a significant increase in the bladder volume when taking doxazosin was observed.

We regarded a decrease of LPP to 50% of starting value as significant, as may be noticed in patients numbered 1 to 4 in Table 1. Similarly, we regarded an increase of LPV 50% up to 200% of starting value as significant in patients 5, 7 and 10.

These significant changes observed in some children call for an evaluation of the impact of selective α1-blockers on the functions of the neurogenic urinary tract using randomized studies in larger groups of patients.

Out of the numerous reported side effects, we have only observed a decrease in blood pressure and the associated drowsiness in one patient from the group under investigation. In one patient, urine continence deterioration was observed, which was confirmed by the follow-up urodynamic tests.

This is similar to reports from other studies showing a low rate of drug related adverse events [10,11,13,14,15,16,17,18,19,20].

For several years, botulinum toxin has been used in cases of neuromuscular disease with muscular hypertension. It is injected into limb muscles in children with spastic cerebral palsy. It is also used to treat torticollis, blepharospasm and in plastic surgery. In the literature, the efficacy of the botulinum toxin in adult patients with both neurogenic and non-neurogenic dysfunction of the urinary tract is well documented. Botulinum toxin was mainly used for injections in the detrusor muscle in order to reduce the pressure inside the bladder in the storage phase [25,26,27].

It was also injected into the sphincter in order to overcome its spasticity and sphincter dyscoordination in the voiding phase [28,29].

Our observations are consistent with the conclusions reached in other studies that showed no evident efficacy of α -blockers in children with neurogenic bladder [18].

The limitation of this study was the small number of children enrolled; therefore, we postulate further research in multicenter, prospective, randomized trials to make the conclusions more reliable.

## 5. Conclusions

Doxazosin did not improve sphincter function parameters in this study group of children with a neurogenic bladder in a statistically significant manner.

## Figures and Tables

**Table 1:** Data from urodynamic and ultrasonographic investigations before and after the treatment.

Patient	Age (Years)	LPP_1_	LPV_1_	LPP_2_	LPV_2_	HDN_1_	HDN_2_
1	2	90	30	45	30		
2	7	135	145	70	145	20	20
3	9	100	230	50	230	30	10
4	4	70	60	30	55		
5	11	60	145	60	225		
6	5	90	70	90	60		
7	14	100	130	95	230	10	10
8	5	55	75	55	75		
9	16	190	290	180	280	30	20
10	6	110	45	120	85	20	20
11	16	210	250	200	250	20	20
12	9	90	140	90	145	20	10
13	5	95	60	100	65		
14	15	170	270				

LPP_1_—leak point pressure (cm H_2_O); LPV_1_—leak point volume (mL); HDN_1_—Hydronephrosis (a-p dimension in mm)—before treatment; LPP_2_, LPV_2_, HDN_2_—after treatment.
